# Transcultural Adaptation and Psychometric Proprieties of the Mental Toughness Inventory for Brazilian Athletes

**DOI:** 10.3389/fpsyg.2021.663382

**Published:** 2021-07-12

**Authors:** Caio Rosas Moreira, Renan Codonhato, Lenamar Fiorese

**Affiliations:** Physical Education Department, State University of Maringá, Maringá, Brazil

**Keywords:** mental toughness, psychometrics, athletes, sport, Brazilian

## Abstract

This study has assessed the psychometric proprieties of the Mental Toughness Inventory (MTI) within the context of Brazilian sports. About 12 professionals participated in the process of adapting and translating the scale to Brazilian Portuguese. Subjects were 575 athletes (23.54 ± 5.79 years old; 58% males) who answered the MTI and the 10-item Connor–Davidson Resilience Scale (CD-RISC-10). Data were analyzed through confirmatory factor analysis (CFA), Cronbach's alpha (α), composite reliability (CR), average variance extracted (AVE), Spearman correlation, and model invariance tests. Results from CFA showed adequate fit for the original 8-item structure of the scale [Chi-square (χ^2^) = 27.041; *p* = 0.078; normalized chi-square (χ^2/df^) = 1.50; comparative fit index (CFI) = 0.988; Tucker–Lewis Index (TLI) = 0.981; root mean square error of approximation (RMSEA) = 0.03 [0.00–0.05]; standardized root mean residual (SRMR) = 0.030] assessing mental toughness (MT) as a single factor and the scale presented satisfactory internal consistency (CR = 0.81; α = 0.82). MT was correlated with resilience (*r* = 0.607), age (*r* = 0.276), and time of experience in the sport (*r* = 0.215). The MTI has also shown partial measurement invariance for sex and complete invariance across sport types. It was concluded that the MTI is a suitable tool for assessing MT in the present sample of Brazilian athletes; this instrument has potential practical application for researchers and sports psychologists who seek to develop the well-being and performance of athletes.

## Introduction

For an athlete to consistently achieve good results, not only physical factors must be trained as psychological aspects are also essential for success. Different mental resources such as context knowledge, emotion regulation, attention regulation, self-efficacy, and optimism are important for an athlete to be prepared for the demands and challenges that are part of competitive sports (Gucciardi et al., 2015). This set of attributes and psychological capacities can be seen as a single aspect: mental toughness (MT).

Defined as a psychological resource, MT is an adaptive mental state characterized by clear goals and efficiency in developing and maintaining goal-directed actions (Gucciardi, 2017). Thus, MT plays an important role for an athlete to consistently perform at high levels despite the stress, challenges, and everyday adversities that are deemed necessary to be developed by athletes who seek to achieve their best results (Weinberg, 2013). In this sense, assessing the MT of athletes is crucial, since it encompasses aspects considered to be essential for success (Gould et al., 1987; Jones et al., 2002; Anthony et al., 2020).

The theoretical comprehension of MT has been described as complex to be structured (Gucciardi and Hanton, 2016). This is partially due to the different forms in which it is measured (uni or multidimensional), the lack of a solid theoretical framework, and the variety of instruments available for assessment (Gucciardi and Gordon, 2011), which have presented certain psychometric inconsistencies for MT measurement (Gucciardi, 2012; Gucciardi et al., 2012). In face of these issues, Gucciardi et al. (2015) have proposed a model that involves the central aspects of MT and presents both theoretical and psychometric rigor for its development.

This theoretical model of MT seeks to relate cognitive, emotional, and motivational domains that are part of the achieving process of an individual, encompassing seven main personal resources: generalized self-efficacy, attention regulation, emotion regulation, success mindset, context knowledge, buoyancy (ability to adapt and perform under pressure), and an optimistic style. Based on this theory, the Mental Toughness Inventory (MTI) was developed and tested across a variety of different contexts which have attested its theoretical and statistical soundness (Gucciardi et al., 2015).

The MTI has been used worldwide to assess MT, having already been translated and adapted to Australia, China, Malaysia (Gucciardi et al., 2016b), China (Li et al., 2019), South Africa (Cowden, 2020), Greece and United States (Stamatis et al., 2019). The scale assesses MT at the specific context of the subject (e.g., academic, sports, and work) as a unidimensional factor, being composed of only eight items. Its convenient short structure represents a single “umbrella” concept encompassing various resources that are all considered essential for the optimal performance of athletes (Cowden, 2016, 2017; García and Santana, 2018).

In Brazil, MT studies are still scarce, possibly due to the lack of reliable instruments for its assessment. Despite the current existence of two instruments measuring MT in Portuguese, some limitations still comprise the applicability of these instruments for the study of MT of athletes. For instance, the Sports Mental Toughness Questionnaire has been only translated to the Portuguese language, without testing its psychometric proprieties for Brazilian populations (Molchansky, 2014); and the scale developed by Corrêa (2019) presents important inconsistencies regarding its theoretical framework, which besides the attempt to estipulate dimensions for MT, also finds difficulties regarding its reproducibility and comparability in the international literature, since there is already a more recent, reliable and widely adopted model that has been considered as the preferred form of assessing MT (Gucciardi et al., 2015).

In face of the current gap in the study of MT in Brazilian sports, the present study has the goal of translating the MTI to Brazilian Portuguese and test its psychometric proprieties in athletes from different competitive levels, providing evidence based on internal structure, relationship with other variables, and measurement invariance.

## Materials and Methods

### Transcultural Adaptation of the MTI to Portuguese

The MTI was developed by Gucciardi et al. (2015) to verify the self-perception of MT of an individual for a certain activity. This questionnaire comprises eight items assessing MT in a unidimensional manner, involving seven given characteristics of a tough mind: generalized self-efficacy (item 1), attention regulation (item 2), emotion regulation (item 3), success mindset (item 4), context knowledge (item 5), buoyancy (items 6 and 7), and optimism (item 8). The answers are given by rating the statements in a 7-point Likert scale ranging from (1) “False, 100% of the time” to (7) “True, 100% of the time.” The final score is obtained by calculating the average of all responses, where higher values represent greater indices of MT for the activity in question.

The MTI was translated and culturally adapted by a committee composed of 12 professionals (four fluent translators, six Ph.D.s in the field of Sport Psychology, and two athletes). These professionals agreed to voluntarily participate in this step. Two bilingual individuals have independently translated the scale from English to Portuguese. Then, two other independent translators have performed the step of back-translation, from the new Portuguese version back to English (Vallerand, 1989; Pasquali, 2009). All four individuals have Brazilian Portuguese as their mother language.

All versions of the scale, along with the original instrument, were analyzed and compared by a group of PhD professors/researchers from the field of Sport Psychology, who have experience in translating and adapting psychometric scales, aiming to find the most adequate version for the MTI in Portuguese (Cassep-Borges et al., 2010). Thus, the translated versions were unified, and the semantics of questions were discussed to obtain a sound instrument for the understanding of the targeted athletic population (Vallerand, 1989; Pasquali, 2009). This final version and the original instruments were analyzed by two English-speaking athletes to further assess divergences and avoid misinterpretation of any item.

To assess the evidence based on the internal content of the items of the scale, a group of six Ph.D. professors/researchers from the field of Sport Psychology have independently rated each of the eight items according to their clarity of language and practical pertinence through a provided evaluation sheet (Supplementary Material), also containing the description of MT and fields for observations and suggestions to be included for each item. The obtained results were used to calculate the content validity coefficient (CVC) of the scale, adopting values above 0.8 as acceptable (Hernández-Nieto, 2002). Items were rated on a Likert scale of five points ranging from (1) “Very low clarity/pertinence” to (5) “Very high clarity/pertinence” for each of the two criteria. Language clarity represents how clear the understanding of the item would be for the target population (athletes), while practical pertinence reflects how relevant each item is to identify those aspects of the life of an individual.

Following the content validity step, a pilot study was performed with a group of 40 athletes (Brazilian native Portuguese-speaking athletes aging between 18 and 23 years old) with the goals of (a) assessing the degree of comprehension of the athletes regarding the adapted questionnaire; (b) verify their comprehension of the instructions and rating system of the scale; (c) find possible weaknesses for the questionnaire application; (d) verify the average time required for answering all items. The sample was selected by convenience and counted with athletes from one team sport (football) and one individual sport (swimming), who answered to the MTI and provided feedback, which was used to create evidence based on the answering process, as recommended by the Standards for Educational and Psychological Testing (American Educational Research Association, National Council on Measurement in Education, and American Psychological Association, 2014). An open question was left at the end of the questionnaire to collect any suggestion or observation from these athletes, who were also verbally questioned regarding the process of filling the scale. This pilot study further attested to the adequacy of the Portuguese version of the MTI for sports, with no doubts being presented by the athletes, who suggested no need for changes in any item of the inventory.

### Participants

This study is part of an institutional project approved by the State University of Maringá Permanent Ethics Committee of Research with Human Beings (Opinion n° 4.022.246). Athletes were given orientations from the authors personally before answering the questionnaire and were provided with information about the research purpose. The answering time was 15 min on average and responses were given individually using pen and paper. The athletes answered the MTI (Gucciardi et al., 2015), the 10-item Connor–Davidson Resilience Scale (CD-RISC-10; Campbell-Sills and Stein, 2007), and a personal information sheet (e.g., age, gender, sport, competitive level, and time of experience).

A total of 575 (23.54 ± 5.21 years old) athletes agreed to participate and have properly filled the scales, being 333 (57.9%) males, 238 (41.4%) females, and four (0.7%) athletes did not answer this question. These athletes represent at a variety of team sports (basketball, futsal, handball, rugby, volleyball, and beach volleyball) and individual sports (athletics, swimming, judo, table tennis, badminton, cycling, rhythmic gymnastics, karate, and taekwondo), and compete at state (26.3%), national (46.0%), and international (27.7%) levels.

We adopted a non-probability convenience sampling aiming at a minimum number of ≥200 participants, to allow for proper use of robust estimation models for ordinal data with a high number of indicators per factor, such as MLR and weighted least squares mean and variance adjusted (WLSMV; Kyriazos, 2018). Inclusion criteria were to agree to voluntarily participate, reading and signing an informed consent term, and being enrolled in the state, national, or international competitions. Cases that did not answer the questionnaires appropriately were excluded.

### Data Analysis

Sample characteristics were reported through descriptive statistics (median, interquartile range, and frequency distribution). All analyses were performed using R software (R Core Team, 2014).

#### Evidence Based on the Internal Structure

Confirmatory factor analysis (CFA) was conducted to test the internal structure of the scale. The Mardia test showed a violation of multivariate normality, thus, the models were estimated using the WLSMV method. The following indicators were used to assess model fitness (Kline, 2015): Chi-square (*X*^2^; *p* > 0.05), normalized chi-square (χ^2/df^ < 3.00), Tucker–Lewis Index (TLI > 0.95), comparative fit index (CFI > 0.95), root mean square error of approximation (RMSEA < 0.08), and standardized root mean residual (SRMR < 0.08). The average variance extracted (AVE) was also calculated, considering values above 0.50 as a satisfactory indicator of construct validity (Hu and Bentler, 1999; Hair et al., 2006). The internal consistency of items was assessed through Cronbach's alpha (α), McDonald's omega (ω), and composite reliability (CR), adopting values above 0.70 as acceptable for all indicators (DeVellis, 2003).

#### Evidence Based on the Relationship With Other Variables

Spearman correlation test was performed to correlate the MT of athletes and resilience scores. Resilience was assessed using the CD-RISC-10 (Campbell-Sills and Stein, 2007), adapted and suitable for Brazilians by Lopes and Martins (2011). This scale is composed of 10 items that are answered on a Likert scale of five points, ranging from 0 to 4, which provides a unidimensional score of resilience.

Moderate to strong correlations between MT and resilience were previously reported in the literature (Gucciardi et al., 2015; Cowden et al., 2016), supporting a certain level of convergence for these two variables. Moreover, since MT is a resource built and learned throughout life, correlations were also calculated for age and time of experience in the sport. We hypothesize that these three variables (resilience, age, and experience) will be positively correlated with the MT scores of Brazilian athletes.

#### Evidence Based on the Measurement Invariance

As the next step within CFA, MTI model invariance was assessed based on the recommendations of Van de Schoot et al. (2012) to test the configural, metric, and scalar invariance of the scale according to sex (males and females) and type of sports (team and individual). Multigroup models were independently estimated based on the two groups of sex and sport to test the configural invariance. Then, factor loadings (FL) were constricted to be equal across groups to test the metric invariance. To establish scalar invariance, both FL and intercepts were constricted. Finally, a full uniqueness model was also estimated, constricting FL, intercepts, and residuals to be equal across groups. Following significant decreases in model fit after imposing model constrictions, subsequent analyses were performed to release variant items and establish partial measurement invariance.

## Results

### Translation and Transcultural Adaptation

The Portuguese version of the MTI that resulted from the translation and back-translation process is presented in Table 1. Items have presented satisfactory CVC for language clarity (0.93) and practical pertinence (1.00) for a total CVC of 0.97 for the scale as a whole. These results suggest that the Brazilian Portuguese version of the MTI can be accurately interpreted by respondents and has practical relevance for the context of sports. This version was named “Inventário de Robustez Mental.”

**Table 1:** Portuguese and English versions of the Mental Toughness Inventory (MTI).

**Escala de Robustez Mental**	**Mental toughness inventory**
1. Eu acredito na minha habilidade para atingir minhas metas.	*(I believe in my ability to achieve my goals)*.
2. Eu sou capaz de regular meu foco quando estou realizando tarefas.	*(I am able to regulate my focus when performing tasks)*.
3. Eu sou capaz de usar minhas emoções para realizar a tarefa da forma que eu quero.	*(I am able to use my emotions to perform the way I want to)*.
4. Eu me esforço para o sucesso contínuo.	*(I strive for continued success)*.
5. Eu utilizo meu conhecimento para atingir minhas metas.	*(I execute my knowledge of what is required to achieve my goals)*.
6. Eu supero constantemente as adversidades.	*(I consistently overcome adversity)*.
7. Eu sou capaz de executar habilidades ou conhecimentos apropriados quando desafiado.	*(I am able execute appropriate skills or knowledge when challenged)*.
8. Eu consigo encontrar algo positivo na maioria das situações.	*(I can find a positive in most situations)*.

### Sample Characteristics

From the total of 575 subjects who agreed to participate, seven had to be excluded due to incorrect filling of the questionnaires. Screening the data for outliners, the Mahalanobis distance suggested the exclusion of another 12 cases, resulting in a final sample of 554 athletes with an average age of 23.6 ± 5.4 years (58.3% males) who had an average time of experience in their sports of 9.7 ± 5.7 years. The majority of athletes trained for individual sports (60.6%), including athletics (*n* = 70), swimming (*n* = 65), karate (*n* = 44), judo (*n* = 37), table tennis (*n* = 33), taekwondo (*n* = 30), badminton (*n* = 26), cycling (*n* = 25), and rhythmic gymnastics (*n* = 6); team sports (39.4%) were represented by athletes from volleyball (*n* = 59), basketball (*n* = 41), rugby (*n* = 39), futsal (*n* = 34), handball (*n* = 27), and beach volleyball (*n* = 18). Competitive levels were as follows: state (*n* = 146; 26.4%), national (*n* = 254; 45.8%), and international (*n* = 154; 27.8%). Nearly half of these athletes (52.9%) were receiving some sort of financial support or sponsorship to train and compete in their sports.

### Evidence Based on the Response Process

**Figure 1:**
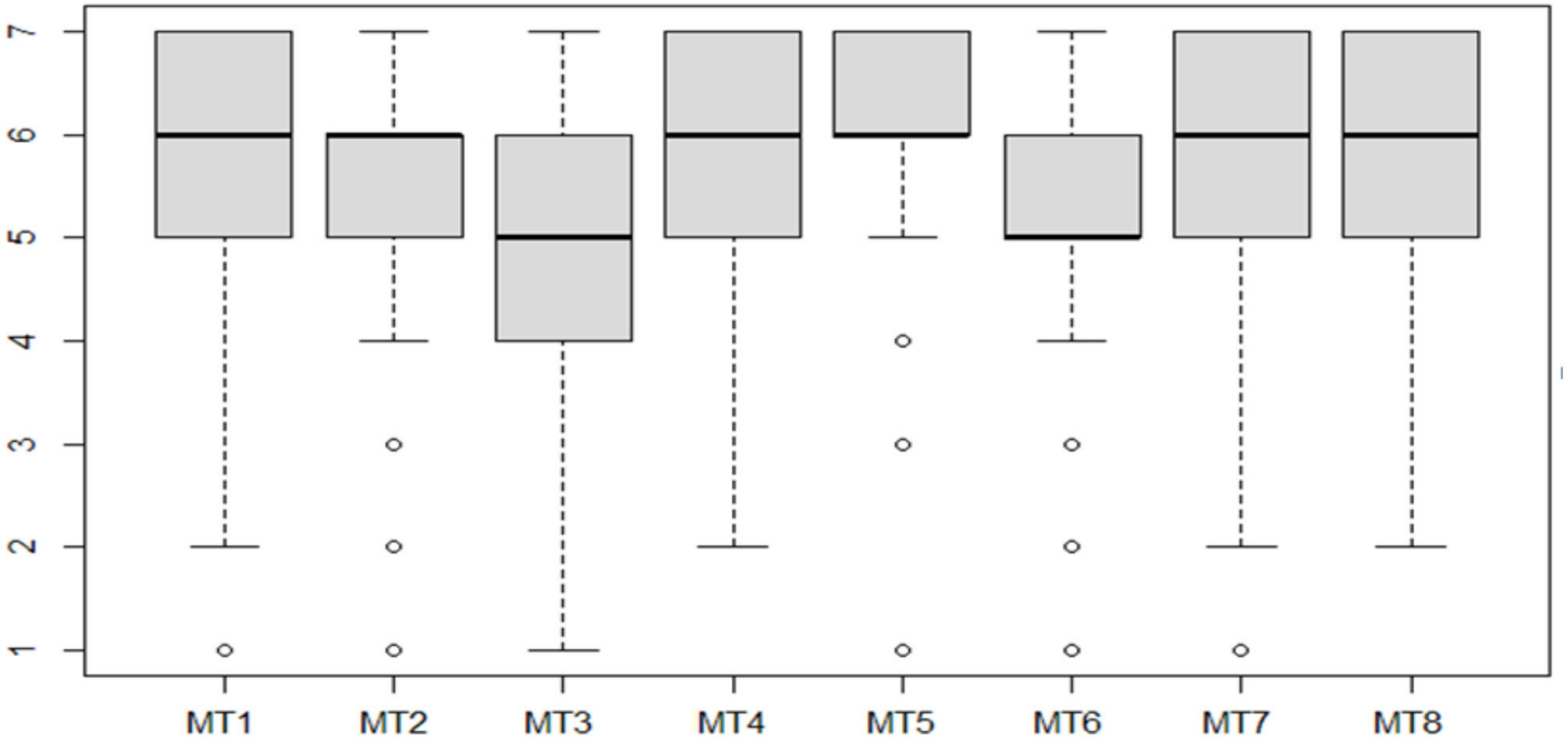
Boxplot of Mental Toughness Inventory (MTI) item scores.

**Figure 2:**
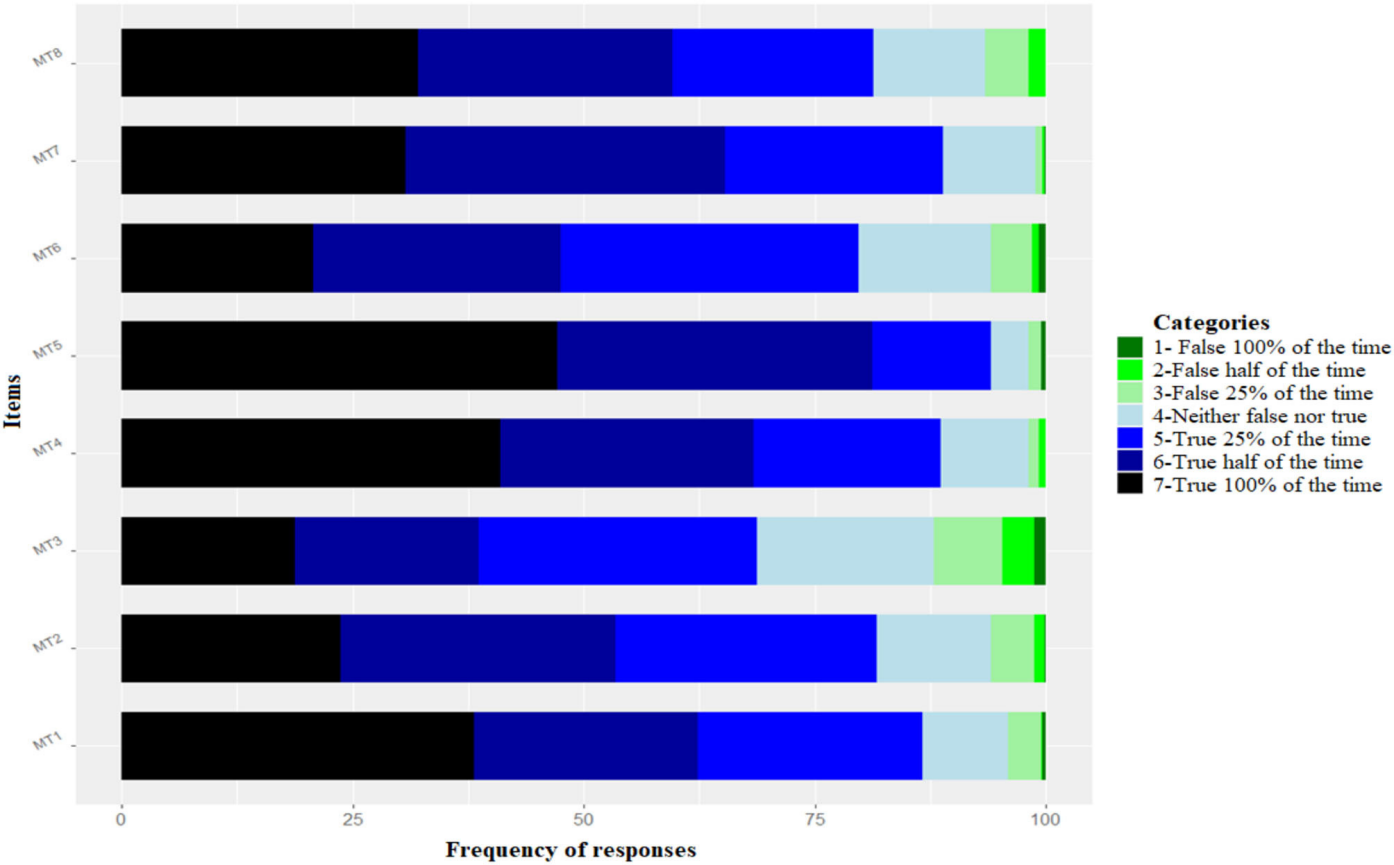
Representation of the Likert scale frequency of responses for the MTI.

Median scores for all items (Figure 1) were above five out of seven points, with item 5 presenting the highest values (Md = 6.0; Q1–Q3 = 6.0–7.0) and item 3 having the lower values (Md = 5.0; Q1–Q3 = 4.0–6.0). It was also observed that the majority of items were most frequently rated from 4 to 7 (Figure 2).

### Evidence Based on Internal Consistency and Reliability

Item correlation matrix (Table 2) suggested that a single factor could explain all eight items since they were all correlated (*p* < 0.001), besides, no correlations were too low or too high, a possible indication of items singularity. These results support a good level of relationship between items (Dima, 2018) and agree with the original theoretical model of the scale (Gucciardi et al., 2015).

**Table 2:** The distribution of MTI items, omega and correlation matrix of items, mental toughness (MT) total score, and external references of age, experience, and resilience for a sample of Brazilian athletes (*n* = 554).

	**MT**	**MT1**	**MT2**	**MT3**	**MT4**	**MT5**	**MT6**	**MT7**	**MT8**
MT1									
MT2		0.312^**^							
MT3		0.354^**^	0.462^**^						
MT4		0.364^**^	0.279^**^	0.230^**^					
MT5		0.384^**^	0.327^**^	0.323^**^	0.525^**^				
MT6		0.331^**^	0.294^**^	0.310^**^	0.370^**^	0.385^**^			
MT7		0.272^**^	0.323^**^	0.291^**^	0.236^**^	0.386^**^	0.408^**^		
MT8		0.322^**^	0.314^**^	0.312^**^	0.260^**^	0.293^**^	0.343^**^	0.323^**^	
MT	ω **=** 0.79	0.642^**^	0.645^**^	0.666^**^	0.593^**^	0.644^**^	0.666^*^	0.605^**^	0.610^**^
Age	0.276^**^	0.199^**^	0.224^**^	0.180^**^	0.102^*^	0.086	0.134^**^	0.134^**^	0.229^**^
Experience	0.215^**^	0.144^**^	0.159^**^	0.149^**^	0.085^*^	0.136^**^	0.155^**^	0.120^**^	0.078
Resilience	0.607^**^	0.411^**^	0.425^**^	0.408^**^	0.323^**^	0.312^**^	0.395^**^	0.354^**^	0.493^**^
Skewness		−0.87	−0.61	−0.49	−1.01	−1.72	−0.68	−0.75	−0.84
Kurtosis		3.34	2.96	2.86	3.93	7.09	3.62	3.52	3.16

* *p < 0.05*;

** *p < 0.01*;

*MT, Mental Toughness total score*;

*ω, McDonald's omega*.

The first model (M1) showed acceptable fit and FL above the recommended cut point (FL > 0.50; *p* < 0.001; Kline, 2015). However, model *p*-value, CFI, and TLI were under the ideal, and modification indices suggested a covariance between items 2 and 3, which were added for testing in the following model (M2). Model fit improved after the modification, but modification indices showed that another improvement could be made by adding a covariance between items 4 and 5. This final model (M3) presented the highest fit and attended to all criteria for model adequacy (Table 3). Reliability measurements were also satisfactory (α = 0.82; ω = 0.79; CR = 0.81), yet values of AVE (0.35) were below the recommended value.

**Table 3:** Model fit indices for the Brazilian MTI (*n* = 554).

	***X^**2**^ (df)***	***p*-value**	***X^**2**^*/df**	**CFI**	**TLI**	**RMSEA [90% C.I.]**	**SRMR**
M1	68.778 (20)	0.001	3.44	0.933	0.906	0.07 [0.05–0.08]	0.048
M2	43.564 (19)	0.001	2.29	0.966	0.950	0.05 [0.03–0.07]	0.039
M3	27.041 (18)	0.078	1.50	0.988	0.981	0.03 [0.00–0.05]	0.030
MTO	39.65 (20)	0.005	1.98	0.980	0.972	0.05 [0.02–0.07]	0.027

*M1, 8-item original model; M2, covariance added between items 2 and 3; M3, covariance added between items 2 and 3, and 4 and 5; MTO, Mental Toughness original study with athletes sample (n = 445) (Gucciardi et al., 2015). χ^2^, Chi-square; df, degrees of freedom; χ^2^/df, normalized chi-square; CFI, comparative fit index; TLI, Tucker–Lewis Index; RMSEA, root mean error of approximation; SRMR, standardized root mean residuals*.

**Figure 3:**
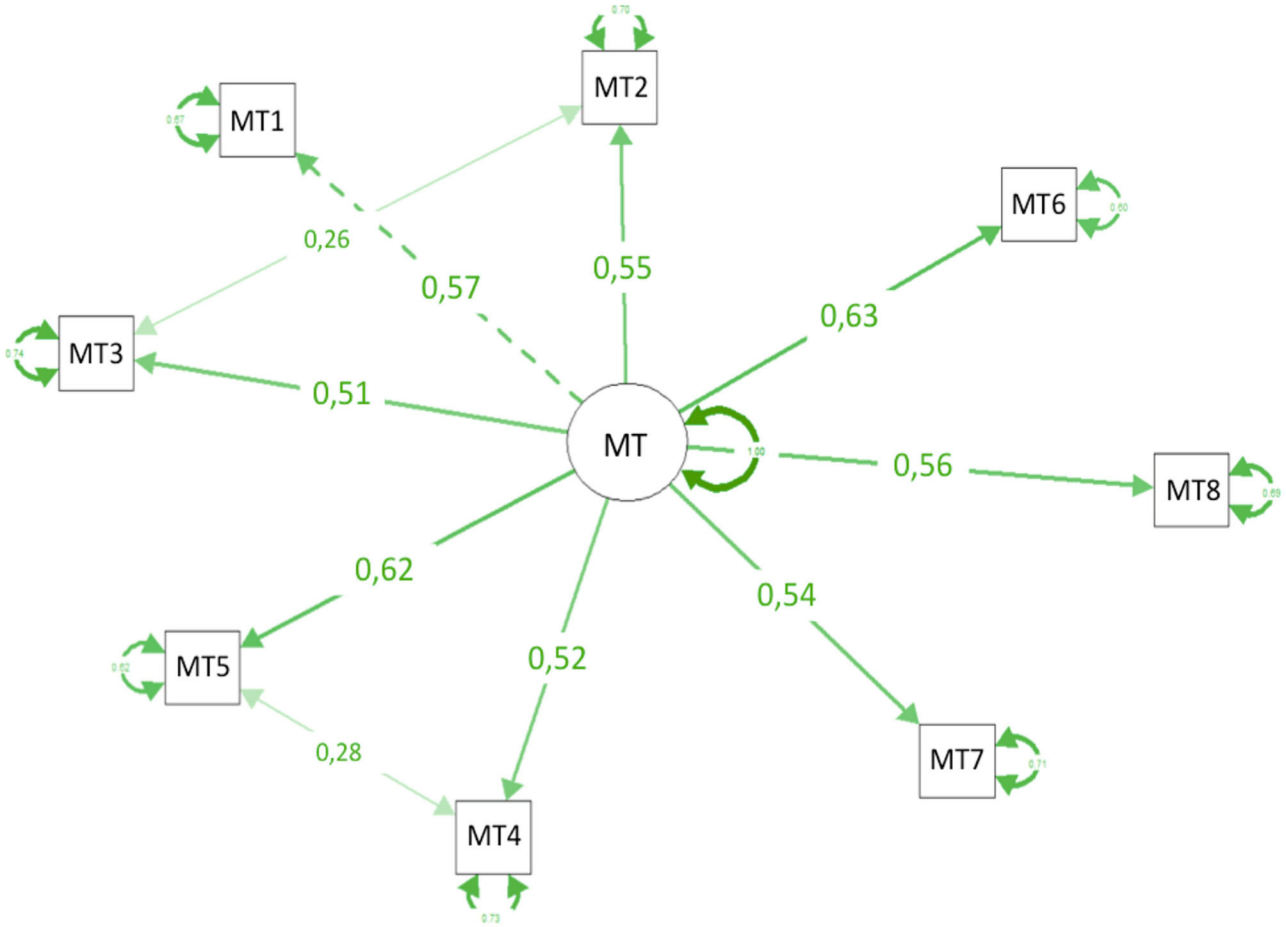
Final confirmatory factor analysis (CFA) model for the MTI in Brazilian sports (coefficients are standardized).

The final model for the Brazilian MTI for sports is presented in Figure 3. The original 8-item structure was confirmed and, considering that spatial distribution of items in the figure is determined by an algorithm, it is possible to observe that items were equally distributed around the latent factor and not too close or too far from each other, suggesting no collinearity between them.

### Evidence Based on the Relationship With Other Variables

Correlation analysis shown in Table 2 revealed that all MTI items were positively correlated to resilience scores (*r* range from 0.312 to 0.493; *p* < 0.01), besides, the total MT score presented a moderate and positive relationship with resilience (*r* = 0.607; *p* < 0.01). Confirming our hypothesis, age (*r* = 0.276) and sports experience (*r* = 0.215) were also positively correlated to MT (*p* < 0.01).

### Evidence Based on Measurement Invariance

Model invariance was assessed for sex and type of sports (Table 4). Model 1 has confirmed that the configural structure is invariant across male and female athletes, and across individual and team sports. Model 2 tested the metric invariance and resulted in a significant decrease in model fit for sex groups, meanwhile, metric invariance was established based on the type of sports. Seeking to establish partial invariance between males and females, FL from items 3 and 8 were released, which improved the model to satisfactory levels. The following step (Model 3) constrained FL and intercepts across items, resulting in a decrease in fit for the model based on sex, and attesting satisfactory scalar invariance between the two sports types. It was possible to establish partial scalar invariance for sex by releasing intercepts of items 1, 3, and 8. Finally, FL, intercepts, and residuals were constrained to test a full uniqueness model, this last model yielded satisfactory results for groups of sex and sports, with partial invariance confirmed for sex, with items 1, 3, and 8 varying between males and females.

**Table 4:** Mental Toughness Inventory measurement invariance models for a sample of Brazilian athletes (*n* = 554).

**Model**	**X^**2**^(df)**	**p**	**X^**2**^/df**	**CFI**	**TLI**	**RMSEA**
**Sex**						
1-Configural	53.250 (36)	0.032	1.48	0.976	0.963	0.04 (0.01–0.06)
2-Metric (partial)	61.375 (41)	0.021	1.49	0.972	0.961	0.04 (0.02–0.06)
3-Scalar (partial)	65.749 (45)	0.023	1.46	0.971	0.964	0.04 (0.02–0.06)
4-Full uniqueness	79.166 (53)	0.011	1.49	0.964	0.961	0.04 (0.02–0.06)
**Type of sport**						
1-Configural	53.592 (36)	0.030	1.49	0.975	0.961	0.04 (0.01–0.06)
2-Metric	52.360 (43)	0.155	1.22	0.987	0.983	0.03 (0.00–0.05)
3-Scalar	60.565 (50)	0.146	1.21	0.985	0.983	0.03 (0.00–0.05)
4-Full uniqueness	79.115 (58)	0.034	1.36	0.970	0.971	0.04 (0.01–0.06)

*Notes for Sex models: Chi-square difference between models: 1 and 2 ($\Delta_{X}^{2}$, p = 0.15), 1 and 3 ($\Delta_{X}^{2}$, p = 0.16), 1 and 4 ($\Delta_{X}^{2}$, p = 0.09)*.

*Notes for Type of Sport models: Chi-square difference between models: 1 and 2 ($\Delta_{X}^{2}$, p = 0.60), 1 and 3 ($\Delta_{X}^{2}$, p = 0.55), 1 and 4 ($\Delta_{X}^{2}$, p = 0.22)*.

## Discussion

The present study had the goal of adapting the MTI for Portuguese-speaking athletes and analyzing its psychometric proprieties in a Brazilian sample of athletes to provide a series of evidence of validity and reliability of the data. This is the first study to assess the psychometric proprieties of the MTI for Brazilian sports. Present results supported the Portuguese version of the MTI for Brazilian athletes across all of the analyses, attesting its internal structure, reliability, measurement invariance, and expected relationship with other variables.

The MTI original structure (i.e., unifactorial with eight items, Gucciardi et al., 2015) was confirmed in the present sample of athletes. All items showed satisfactory FL and were moderately correlated with each other and the total MT score, indicating the relationship of items with the global concept of MT (Dima, 2018). In face of recent critics of the use of Cronbach's α in the psychometric literature (Padilla and Divers, 2016), we have also calculated the CR of the scale, which also presented adequate values (CR > 0.70).

The first model (M1, Table 3) presented an acceptable fit (RMSEA < 0.08; CFI > 0.90; TLI > 0.90; SRMR < 0.08) for a model with all eight items presenting FL over 0.50 for a single factor of MT (Hair et al., 2006). Still, two modifications were subsequently performed to achieve an ideal fit, adding covariances between items 2 (attention regulation) and 3 (emotion regulation), and items 4 (success mindset) and 5 (context knowledge). This is the first psychometric study of the MTI to report covariances between these items. However, such covariances are likely to be due to the inherent relationship between emotion and attention regulation (items 2 and 3) and using the knowledge of the environment while pursuing success (items 4 and 5), and not due to collinearity between items. It is worth noting that these covariances were relatively weak (0.26 and 0.28, respectively), and the FL of the items remained above 0.50. Moreover, the improved model showed a satisfactory fit (M3, Table 3).

A limitation was found for the AVE of the scale of 0.35, which stood below the recommended value of >0.50. Such a result could be due to the low variance of responses for all items (Figures 1, 2), which mostly varied within the upper portion of the scale (items 5, 6, and 7). As a consequence, the majority of the evidence was gathered from individuals with moderate to high levels of MT, while little evidence was available regarding the individuals scoring low on this attribute (MT < 4.00). Studies have shown that the involvement with sports may promote MT (Gucciardi et al., 2016a; García and Santana, 2018; Zeiger and Zeiger, 2018), which was also seen in the results (Table 2) along with a positive correlation with age and time of sporting experience. Developing a robust mind is linked to several aspects related to the process, the time involved, the self-perception of the subject, and the context in which the individual finds him or herself (Anthony et al., 2016). Considering the average age of the sample (23.6 ± 5.4 years) and time of experience in their sports (9.7 ± 5.7 years), the majority of the sample may have developed considerable levels of MT due to sports. Once MT is a personal component developed over time, age and time of experience contribute together to a more robust mind (Cowden, 2017). However, more studies are still required to support this hypothesis. Still, it is also suggested that future studies include a wider representation of individuals scoring below four points for MT.

Besides its relationship with age and sports experience, MT was strongly associated with psychological resilience as well (Table 2). Both resilience and MT represent a strengthening of the mind throughout life, a relationship that has already been reported by other studies (Nicholls et al., 2009; Cowden et al., 2016) and offers support for the convergent validity of MTI for Brazilian sports.

Testing the measurement invariance of the scale (Table 4), it was possible to observe that the MTI is structured and understood similarly (without divergent interpretations) by athletes from individual and team sports. On the other hand, only partial measurement invariance was established according to sex, showing that male and female athletes have attributed different importance for some of the items. For instance, item 3 (“I am able to use my emotions to perform the way I want to”) had a higher contribution for the MT of male athletes (FL = 0.53) than it did for female athletes (FL = 0.48), meanwhile, item 8 (“I can find a positive in most situations”) loaded more strongly for female (FL = 0.64) rather than male athletes (FL = 0.47; Supplementary Material). Such a result was expected (Nicholls et al., 2009; Crust and Keegan, 2010; Zeiger and Zeiger, 2018) considering how MT might be influenced by social idealizations of male masculinity in competitive sports (Gucciardi et al., 2017).

Although the study design sought to include a heterogeneous sample comprising athletes from different levels and sports, there was still a limitation on the understanding of low-MT athletes. Due to the cross-sectional nature of the investigation, which is limited to the self-perception of the subject at that given moment, and the contextual characteristic of the selected sample, composed of athletes at the final stage of the competition, the majority of the evidence was predominantly obtained from athletes scoring at the upper portion of the scale, leading to AVE values below ideal. Another limitation was the data collection at a single time point, which does not offer evidence of the temporal stability of the scale.

It is understood that the validation of a psychometric scale is an ongoing process, in this sense; we would like to encourage future studies adopting the MTI for Brazilian athletes to replicate the findings and offer further evidence of its applicability. Moreover, future studies should consider including younger athletes and athletes at the initiation level to look for more evidence on the lower portion of the scale and test the influence of sports over the levels of MT of an individual. Longitudinal studies are also suggested for improved comprehension of the Sport x MT relationship. Considering that the recruited sample was part of a competition involving a single state from the south of Brazil, more studies are also necessary to assess possible regional differences across the country or even differences among other Portuguese-speaking nations.

Finally, the present version of the MTI has shown to be a reliable tool for assessing MT levels of the present sample of Brazilian athletes. The results will contribute to MT research in Brazilian sports, which can allow researchers and professionals working with sports to draw interventions, investigations, and approaches based on evidence from their specific context. The work on improving the MT of athletes should be an essential part of the psychological training of athletes, once is highly advisable for those who seek better performances in sports and life.

## Data Availability Statement

The original contributions presented in the study are included in the article/Supplementary Material, further inquiries can be directed to the corresponding author/s.

## Ethics Statement

The studies involving human participants were reviewed and approved by Ethics Committee of State University of Maringá. The patients/participants provided their written informed consent to participate in this study.

## Author Contributions

All authors contributed to the conception and design of the study, reviewed the document, approved the final version for submission, and commented on previous versions of the manuscript. Material preparation, data collection, and analysis were performed by CM and RC. The first draft of the manuscript was written by CM.

## Conflict of Interest

The authors declare that the research was conducted in the absence of any commercial or financial relationships that could be construed as a potential conflict of interest.

## References

[B1] American Educational Research Association National Council on Measurement in Education, and American Psychological Association. (2014). Standards for Educational and Psychological Testing. American Educational Research Association.

[B2] Anthony D. R. Gordon S. Gucciardi D. F. (2020). A qualitative exploration of mentally tough behaviour in Australian football. J. Sports Sci. 3, 308–319. 10.1080/02640414.2019.1698002 31783717

[B3] Anthony D. R. Gucciardi D. F. Gordon S. (2016). A meta-study of qualitative research on mental toughness development. Int. Rev. Sport Exerc. Psychol. 9, 160–190. 10.1080/1750984X.2016.1146787

[B4] Campbell-Sills L. Stein M. B. (2007). Psychometric analysis and refinement of the connor-davidson resilience scale (CD-RISC): validation of a 10-item measure of resilience. J. Traum. Stress 20, 1019–1028. 10.1002/jts.20271 18157881

[B5] Cassep-Borges V. Balbinotti M. A. A. Teodoro M. L. M. (2010). Translation of Content Validation: A Proposal for Adaptation of Instruments. Pasqualli L, Organizer. Psychological Instrumentation. Porto Alegre: Artmed, 506–520.

[B6] Corrêa M. F. (2019). Propriedades psicométricas da escala de robustez mental no esporte para atletas brasileiros adultos (RME-A) (Master's thesis). Universidade São Judas Tadeu, São Paulo, Brazil.

[B7] Cowden R. G. (2016). Competitive performance correlates of mental toughness in tennis: a preliminary analysis. Percept. Motor Skills 123, 341–360. 10.1177/0031512516659902 27502244

[B8] Cowden R. G. (2017). Mental toughness and success in sport: a review and prospect. Open Sports Sci. J. 10, 1–14. 10.2174/1875399X01710010001

[B9] Cowden R. G. (2020). Mental Toughness Inventory: factorial validity and ethnic group measurement equivalence in competitive tennis. Curr. Psychol. 39, 736–741. 10.1007/s12144-018-9798-6

[B10] Cowden R. G. Meyer-Weitz A. Oppong Asante K. (2016). Mental toughness in competitive tennis: relationships with resilience and stress. Front. Psychol. 7:320. 10.3389/fpsyg.2016.00320 27014132 PMC4791384

[B11] Crust L. Keegan R. (2010). Mental toughness and attitudes to risk-taking. Pers. Individ. Dif. 49, 164–168. 10.1016/j.paid.2010.03.026

[B12] DeVellis R. F. (2003). Scale Development: Theory and Applications. Thousand Oaks, CA: Sage.

[B13] Dima A. L. (2018). Scale validation in applied health research: tutorial for a 6-step R-based psychometrics protocol. Health Psychol. Behav. Med. 6, 136–161, 10.1080/21642850.2018.1472602 34040826 PMC8133536

[B14] García F. G. Santana J. (2018). Exploring mental toughness in soccer players of different levels of performance. Rev. Iberoam Psicol. Ejerc. Dep. 13, 297–303.

[B15] Gould D. Hodge K. Peterson K. Petlichkoff L. (1987). Psychological foundations of coaching: similarities and differences among intercollegiate wrestling coaches. Sport Psychol. 1, 293–308. 10.1123/tsp.1.4.293

[B16] Gucciardi D. F. (2012). Measuring mental toughness in sport: a psychometric examination of the Psychological Performance Inventory-A and its predecessor. J. Pers. Assess. 94, 393–403. 10.1080/00223891.2012.660292 22369040

[B17] Gucciardi D. F. (2017). Mental toughness: progress and prospects. Curr. Opin. Psychol. 16, 17–23. 10.1016/j.copsyc.2017.03.010 28813344

[B18] Gucciardi D. F. Gordon S. (eds.). (2011). Mental Toughness in Sport: Developments in Theory and Research,. London, UK: Routledge. 10.4324/9780203855775

[B19] Gucciardi D. F. Hanton S. (2016). “Mental toughness: critical reflections and future considerations,” in The Routledge International Handbook of Sport Psychology, eds Schinke R. McGannon K. Smith B. (New York, NY: Routledge), 439–448.

[B20] Gucciardi D. F. Hanton S. Fleming S. (2017). Are mental toughness and mental health contradictory concepts in elite sport? A narrative review of theory and evidence. J. Sci. Med. Sport. 20, 307–311. 10.1016/j.jsams.2016.08.006 27568074

[B21] Gucciardi D. F. Hanton S. Gordon S. Mallett C. J. Temby P. (2015). The concept of mental toughness: tests of dimensionality, nomological network and traitness. J. Pers. 83, 26–44. 10.1111/jopy.12079 24428736

[B22] Gucciardi D. F. Hanton S. Mallett C. J. (2012). Progressing measurement in mental toughness: a case example of the Mental Toughness Questionnaire 48. Sport Exerc. Perform. Psychol. 1, 194–214. 10.1037/a0027190

[B23] Gucciardi D. F. Peeling P. Ducker K. J. Dawson B. (2016a). When the going gets tough: mental toughness and its relationship with behavioural perseverance. J. Sci. Med. Sport 19, 81–86. 10.1016/j.jsams.2014.12.005 25554654

[B24] Gucciardi D. F. Zhang C.-Q. Ponnusamy V. Si G. Stenling A. (2016b). Cross-cultural invariance of the Mental Toughness Inventory among Australian, Chinese and Malaysian athletes: a Bayesian estimation approach. J. Sport Exerc. Psychol. 38, 187–202. 10.1123/jsep.2015-0320 27390084

[B25] Hair J. F. Black B. Babin B. Anderson R. E. Tatham R. L. (2006). Multivariate Data Analysis, 6th Edn. Upper Saddle River, NJ: Pearson Prentice Hall.

[B26] Hernández-Nieto R. A. (2002). Contributions to Statistical Analysis. Mérida: Universidad de Los Andes.

[B27] Hu L. T. Bentler P. M. (1999). Cutoff criteria for fit indexes in covariance structure analysis: conventional criteria versus new alternatives. Struct. Equ. Model. 6, 1–55. 10.1080/10705519909540118

[B28] Jones G. Hanton S. Connaughton D. (2002). What is this thing called mental toughness? An investigation of elite sport performers. J. Appl. Sport Psychol. 14, 205–218. 10.1080/10413200290103509

[B29] Kline R. B. (2015). Principles and Practice of Structural Equation Modeling, 4th Edn. New York, NY: The Guilford Press.

[B30] Kyriazos T. A. (2018). Applied psychometrics: sample size and sample power considerations in factor analysis (EFA, CFA) and SEM in general. Psychology 9, 2207–2230. 10.4236/psych.2018.98126

[B31] Li C. Zhang C. Zhang L. (2019). Further examination of the psychometric properties of the mental toughness inventory: evidence from Chinese athletes and university students. Curr. Psychol. 38, 1328–1334. 10.1007/s12144-017-9692-7

[B32] Lopes V. R. Martins M. D. C. F. (2011). Factorial validation and adaptation of the connor-davidson resilience scale (CD-RISC-10) for brazilians. Rev. Psicol. Organ. Trab. 11, 36–50.

[B33] Molchansky S. (2014). Tradução e adaptação transcultural do Sport Mental Toughness Questionnaire para a língua portuguesa do Brasil (Master's thesis). Faculdade de Educação Física, Universidade Estadual de Campinas, Campinas, Brazil.

[B34] Nicholls A. R. Polman R. C. Levy A. R. Backhouse S. H. (2009). Mental toughness in sport: achievement level, gender, age, experience, and sport type differences. Pers. Individ. Dif. 47, 73–75. 10.1016/j.paid.2009.02.006

[B35] Padilla M. A. Divers J. (2016). A comparison of composite reliability estimators: coefficient omega confidence intervals in the current literature. Educ. Psychol. Meas. 76, 436–453. 10.1177/0013164415593776 29795872 PMC5965559

[B36] Pasquali L. (2009). Instrumentação Psicológica: Fundamentos e Práticas. Porto Alegre: Artmed Editora.

[B37] R Core Team (2014). R: A Language and Environment for Statistical Computing. Vienna: R Foundation for Statistical Computing.

[B38] Stamatis A. Morgan G. B. Papadakis Z. Mougios V. Bogdanis G. Spinou A. (2019). Cross-cultural invariance of the Mental Toughness Index among American and Greek athletes. Curr. Psychol. 1–8. 10.1007/s12144-019-00532-2

[B39] Vallerand R. J. (1989). Vers une méthodologie de validation trans-culturelle de questionnaires psychologiques: implications pour la recherche en langue française. Can. Psychol. 30:662. 10.1037/h0079856 6850979

[B40] Van de Schoot R. Lugtig P. Hox J. (2012). A checklist for testing measurement invariance, Eur. J. Dev. Psychol. 9, 486–492, 10.1080/17405629.2012.686740

[B41] Weinberg R. (2013). Mental toughness: what is it and how to build it. J. Phys. Educ. 24, 1–10. 10.4025/reveducfis.v24i1.17523

[B42] Zeiger J. S. Zeiger R. S. (2018). Mental toughness latent profiles in endurance athletes. PLoS ONE 13:e0193071. 10.1371/journal.pone.0193071 29474398 PMC5825049

