# Supplementary material for: Transcultural Adaptation and Psychometric Proprieties of the Mental Toughness Inventory for Brazilian Athletes
Source: Front Psychol. 2021 Jul 12;12:663382. doi: 10.3389/fpsyg.2021.663382 (PMC8311166; doi:10.3389/fpsyg.2021.663382)
Supplement: Supplementary file 1 [file Table_1.DOCX]

**ORIENTAÇÕES PARA O PREENCHIMENTO DA FICHA DE AVALIAÇÃO** **DO**

***MENTAL TOUGHNESS INDEX* (MTI)**

- “**Clareza de linguagem”:** o(a) senhor(a) acredita que a linguagem de cada item é suficientemente clara, compreensível e adequada para esta população (atletas)? Em que nível?
- **“Pertinência prática”:** o(a) senhor(a) acredita que cada item proposto é pertinente para esta população (atletas)? Em que nível?
- **“Relevância teórica”:** o(a) senhor(a) acredita que o conteúdo do item é representativo do comportamento que se quer medir, o Mental Toughness? Em que nível?
- Gucciardi e col. (2015) conceituam o *Mental Toughness* como uma capacidade individual de produzir consistentemente altos níveis de desempenho subjetivo (ex. metas e esforços pessoais) ou objetivos (ex. vendas, tempo de prova, nota na escola) em relação aos desafios, agentes estressores, e adversidades significativas vivenciadas no cotidiano. Para uma mente robusta (*tough*) é necessário diversos elementos consolidados no indivíduo, considerados elementos chave para o *Mental Toughness* de uma pessoa, sendo sua auto-crença, regulação da atenção, regulação da emoção, sucesso na *Mindset*, conhecimento do contexto inserido, flutuabilidade (capacidade de se adaptar a este contexto), e ser otimista frente as adversidades,

**Legenda**: Escala *Likert*: (1) pouquíssima; (2) pouca; (3) média; (4) muita; (5) muitíssima.

**Planilha de avaliação da validade de conteúdo**

|  | **Item** | **Clareza de linguagem** | **Pertinência prática** | **Relevância teórica** | **Observação** |
| --- | --- | --- | --- | --- | --- |
| 1. | Eu acredito na minha habilidade para atingir minhas metas.  I believe in my ability to achieve my goals. | 1/ 2/ 3/ 4/ 5  ( )( )( )( )( ) | 1/ 2/ 3/ 4/ 5  ( )( )( )( )( ) | 1/ 2/ 3/ 4/ 5  ( )( )( )( )( ) |  |
| 2. | Eu sou capaz de regular meu foco quando estou realizando tarefas.  I am able to regulate my focus when performing tasks. | 1/ 2/ 3/ 4/ 5  ( )( )( )( )( ) | 1/ 2/ 3/ 4/ 5  ( )( )( )( )( ) | 1/ 2/ 3/ 4/ 5  ( )( )( )( )( ) |  |
| 3. | Eu sou capaz de usar minhas emoções para realizar a tarefa da forma que eu quero.  I am able to use my emotions to perform the way I want to. | 1/ 2/ 3/ 4/ 5  ( )( )( )( )( ) | 1/ 2/ 3/ 4/ 5  ( )( )( )( )( ) | 1/ 2/ 3/ 4/ 5  ( )( )( )( )( ) |  |
| 4. | Eu me esforço pelo sucesso contínuo.  I strive for continued success. | 1/ 2/ 3/ 4/ 5  ( )( )( )( )( ) | 1/ 2/ 3/ 4/ 5  ( )( )( )( )( ) | 1/ 2/ 3/ 4/ 5  ( )( )( )( )( ) |  |
| 5. | Eu utilizo meu conhecimento necessário para atingir minhas metas.  I execute my knowledge of what is required to achieve my goals. | 1/ 2/ 3/ 4/ 5  ( )( )( )( )( ) | 1/ 2/ 3/ 4/ 5  ( )( )( )( )( ) | 1/ 2/ 3/ 4/ 5  ( )( )( )( )( ) |  |
| 6. | Eu supero constantemente as adversidades.  I consistently overcome adversity. | 1/ 2/ 3/ 4/ 5  ( )( )( )( )( ) | 1/ 2/ 3/ 4/ 5  ( )( )( )( )( ) | 1/ 2/ 3/ 4/ 5  ( )( )( )( )( ) |  |
| 7. | Eu sou capaz de executar habilidades ou conhecimentos apropriados quando desafiado.  I am able execute appropriate skills or knowledge when challenged. | 1/ 2/ 3/ 4/ 5  ( )( )( )( )( ) | 1/ 2/ 3/ 4/ 5  ( )( )( )( )( ) | 1/ 2/ 3/ 4/ 5  ( )( )( )( )( ) |  |
| 8. | Eu consigo encontrar algo positivo na maioria das situações.  I can find a positive in most situations. | 1/ 2/ 3/ 4/ 5  ( )( )( )( )( ) | 1/ 2/ 3/ 4/ 5  ( )( )( )( )( ) | 1/ 2/ 3/ 4/ 5  ( )( )( )( )( ) |  |
