# Supplementary figures and images for: Transcultural Adaptation and Psychometric Proprieties of the Mental Toughness Inventory for Brazilian Athletes
Source: Front Psychol. 2021 Jul 12;12:663382. doi: 10.3389/fpsyg.2021.663382 (PMC8311166; doi:10.3389/fpsyg.2021.663382)

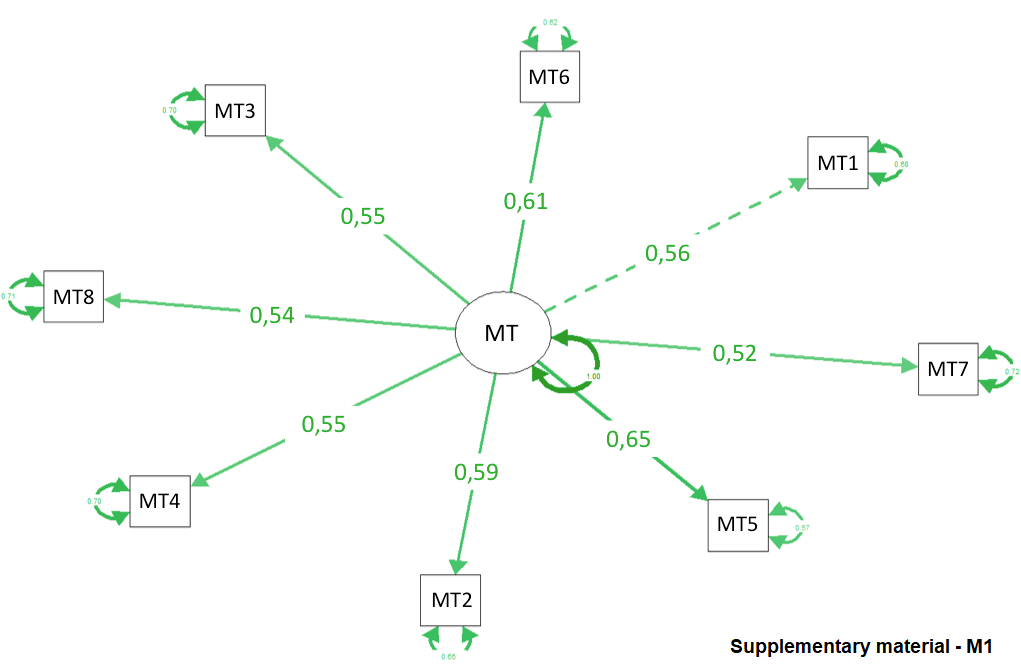

Supplement: Supplementary file 3 [file Image_1.TIF]

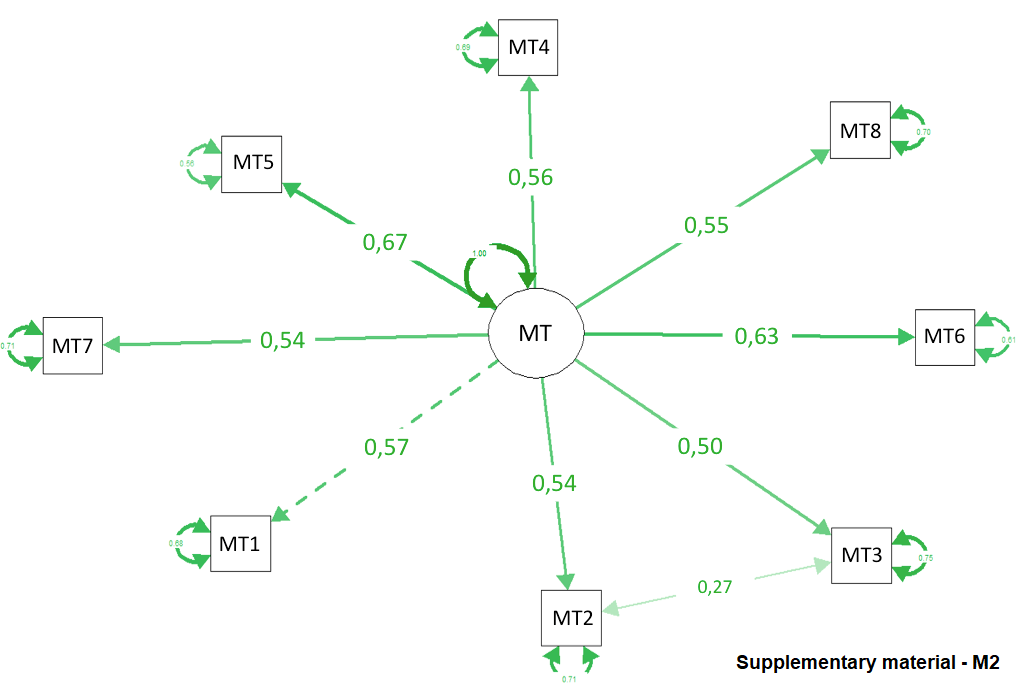

Supplement: Supplementary file 4 [file Image_2.TIF]

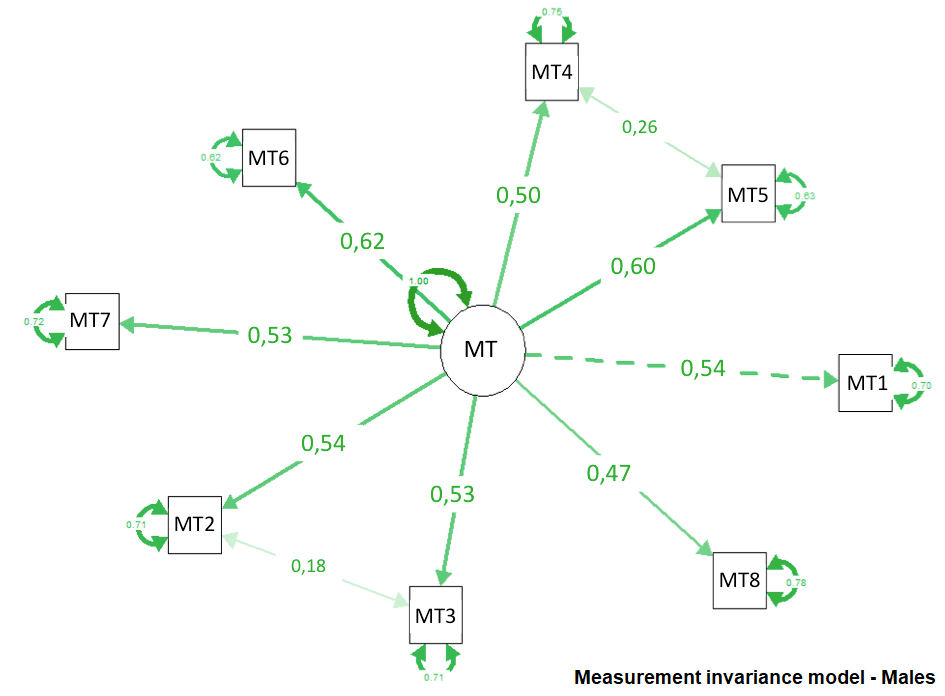

Supplement: Supplementary file 5 [file Image_3.TIF]

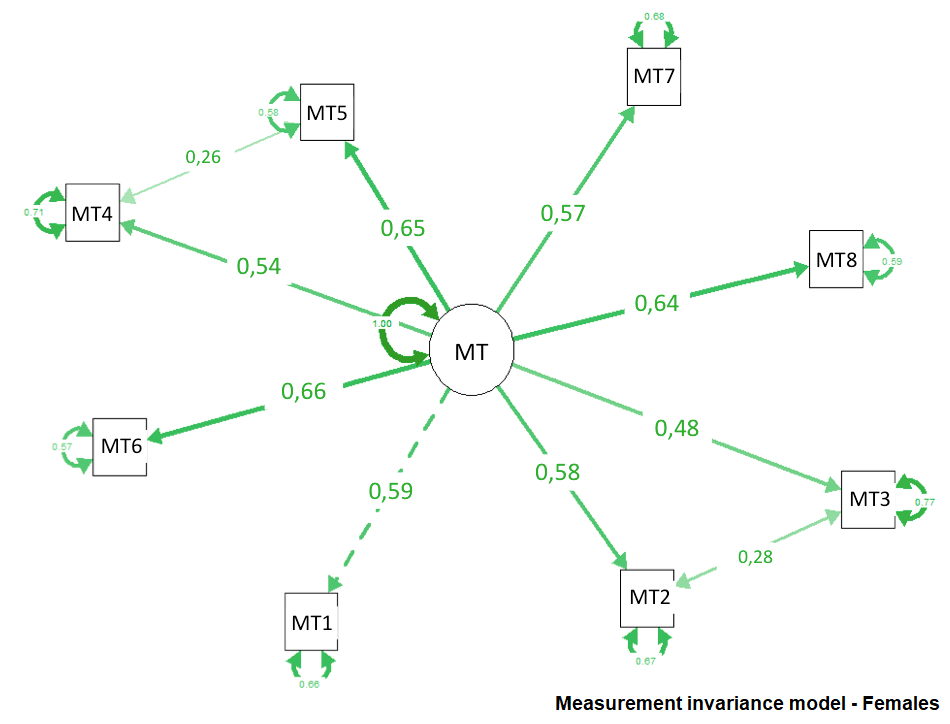

Supplement: Supplementary file 6 [file Image_4.TIF]

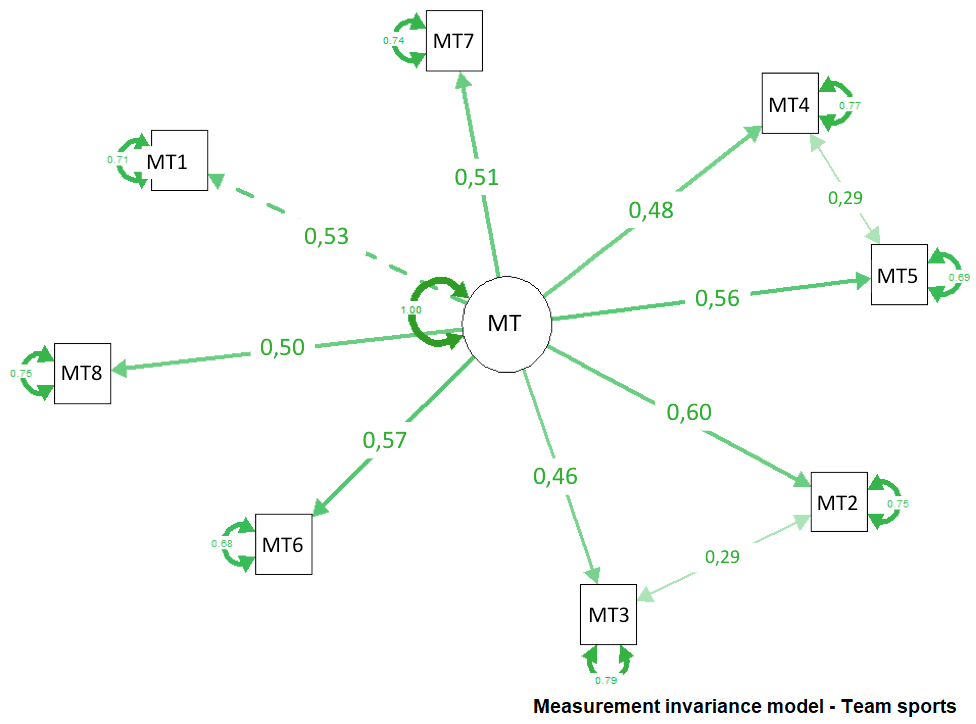

Supplement: Supplementary file 7 [file Image_5.TIF]

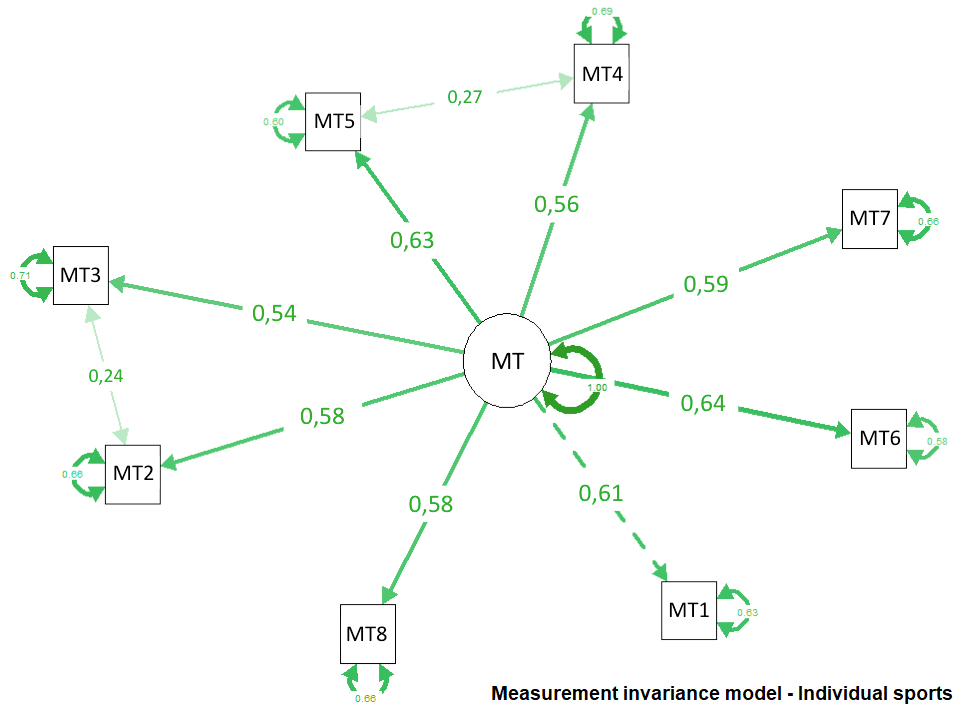

Supplement: Supplementary file 8 [file Image_6.TIF]
